# The Effects of a Food Supplement, Based on Co-Micronized Palmitoylethanolamide (PEA)–Rutin and Hydroxytyrosol, in Metabolic Syndrome Patients: Preliminary Results

**DOI:** 10.3390/nu17030413

**Published:** 2025-01-23

**Authors:** Kevin Cornali, Manuela Di Lauro, Giulia Marrone, Claudia Masci, Giulia Montalto, Alfredo Giovannelli, Carlo Schievano, Manfredi Tesauro, Massimo Pieri, Sergio Bernardini, Annalisa Noce

**Affiliations:** 1 Department of Experimental Medicine, PhD School in Biochemistry and Molecular Biology, University of Rome Tor Vergata, 00133 Rome, Italy; kevin.cornali@students.uniroma2.eu; 2 Department of Systems Medicine, University of Rome Tor Vergata, 00133 Rome, Italy; dilauromanuela@gmail.com (M.D.L.); giulia.marrone@uniroma2.it (G.M.); masciclaudia@gmail.com (C.M.); mtesauro@tiscali.it (M.T.); 3 School of Specialization in Nephrology, University of Rome Tor Vergata, 00133 Rome, Italy; giulia.montalto@ptvonline.it; 4 Unit of Laboratory Medicine, University Hospital Tor Vergata, 00133 Rome, Italy; alfredo.giovannelli@gmail.com (A.G.); massimo.pieri@uniroma2.it (M.P.); 5 Innovative Statistical Research, 35100 Padua, Italy; cs@i-stat.it; 6 Department of Experimental Medicine, University of Rome Tor Vergata, 00133 Rome, Italy; 7 UOSD Nephrology and Dialysis, Policlinico Tor Vergata, 00133 Rome, Italy

**Keywords:** metabolic syndrome, co-micronized palmitoylethanolamide, hydroxytyrosol, rutin, adiposopathy, meta-inflammation, mediterranean diet, natural bioactive compounds, body composition assessment

## Abstract

Background: Metabolic syndrome (MetS) patients have impaired hypothalamic regulatory functions involved in food intake and energy expenditure and suffer from a state of meta-inflammation. Pre-clinical studies demonstrated that ultramicronized palmitoylethanolamide (PEA) acts both on the adipose tissue and the central nervous system, while hydroxytyrosol (HTyr) counteracts several types of dysmetabolism. Objectives: The aim of our randomized crossover double-blind placebo-controlled pilot study was to evaluate the potential effects of a food supplement (FS) containing a co-micronized formulation of PEA and rutin along with HTyr, combined with a tailored calorie-controlled Mediterranean diet, in patients with MetS. Methods: Nineteen patients were enrolled and block-randomized to an eight-week MD together with the FS or placebo. After a two-week washout period, the treatments were reversed. Data on laboratory parameters and those detected by capillary sampling, anthropometry, body composition analysis, ultrasound examination, blood pressure monitoring, the 36-Item Short-Form Health Survey questionnaire, handgrip strength test, and physical performance tests were collected at each time point (protocol code R.S. 262.22, registered on 20 December 2022). Results: At the end of the study, patients supplemented with the FS showed a significant reduction in body weight, body mass index, fat mass, and inflammation biomarkers (CRP and ESR), compared to placebo-supplemented patients. In contrast, the fat-free mass, phase angle, and body cell mass were increased in FS compared to placebo patients. Conclusions: Although preliminary, the results of our clinical study suggest that co-micronized PEA–rutin and HTyr may be of help against adiposopathy in patients with MetS.

## 1. Introduction

Metabolic syndrome (MetS) is defined by the World Health Organization (WHO) as a complex disorder represented by a cluster of interconnected comorbidities, including abdominal obesity, hyperglycemia, arterial hypertension, and dyslipidemia, that increase the risk of cardiovascular atherosclerotic diseases and other metabolic dysfunctions [1]. The accumulation of visceral adipose tissue in fact causes an increase in the production of pro-inflammatory cytokines and a reduction in anti-inflammatory ones, such as adiponectin, predisposing the organism to meta-inflammation. As a consequence, the increased beta cell function and insulin resistance promote impaired control of glucose metabolism, which triggers metabolic disorders [2].

MetS is a worldwide public health problem that dramatically affects human health and reduces patients’ quality of life (QoL) [3].

The incidence of MetS is often linked with that of obesity and of type 2 diabetes mellitus (T2DM). According to data reported by the WHO, obesity affects more than 890 million adults worldwide (13%) [4], while, according to the International Diabetes Federation (IDF), T2DM affects more than 151 million people [5]. The IDF has estimated that 20–25% of adults worldwide have MetS [6]. In the United States, the number of adults with MetS is dramatically increasing, with the rate rising from to 32.5% in 2011 to 36.9% in 2016 [7], while, in Europe, it is estimated that 12–26% of adults suffer from this condition [8].

Economically, a patient with MetS costs about USD 5.477 per year, with an increase in expenditure of USD 2.151 if compared to healthy subjects. The cost rises by up to 24% if a patient has all five criteria defined by the IDF. The prevalence of MetS and its high operating costs show no signs of slowing down, making MetS a complex financial challenge regarding global public health [9].

The causes of MetS include a sedentary lifestyle, unhealthy dietary habits, and environmental and genetic factors [10].

For the clinical management of MetS, polypharmacotherapy is often required. However, the latter can sometimes cause side effects and can be associated with a patient’s reduced compliance [11].

In light of this, secondary prevention requires the modification of patients’ eating habits. Several dietary patterns have been associated with some beneficial effects for the organism, but, in the literature, the largest body of evidence is related to the traditional dietary habits and healthy lifestyle followed by those populations living in the Mediterranean region [12]. In 2010, the United Nations Educational, Scientific and Cultural Organization—UNESCO—proclaimed the Mediterranean diet (MD) as a “World Cultural Heritage”, highlighting its ability to preserve health and improve longevity [13]. The MD’s benefits for human health were first investigated by the MD’s pioneer, the American physiologist Ancel Keys, and by the Italian Flaminio Fidanza with the Seven Country Study, which compared the eating habits of Mediterranean cohorts with those of non-Mediterranean ones. Keys and Fidanza observed that the death rates for all causes and for coronary heart disease were positively related to the average percentage of the dietary energy from saturated fatty acids and negatively with those from monounsaturated ones. In fact, the mortality rates were lower in cohorts that had used extra virgin olive oil (EVOO) as the main fat source [14]. The MD pyramid can also be depicted as a Paestum Temple, whose stylobate is represented by a healthy lifestyle, with energy intake equal to expenditure and the high consumption of EVOO and red wine. The supporting columns are represented by whole grains, legumes, fish, vegetables, and fresh and dried fruit, while the lintel function is provided by meat, dairy products, fats, and sugar, which should be scarcely consumed [15].

Over the years, different scientific evidence has shown that “eating Mediterranean” maintains individuals’ mental and physical well-being, and it represents a protective factor against the onset of cardiovascular and metabolic diseases, cancer, aged-related pathologies, and obesity [16].

These protective effects are mostly due to the combination of vitamins (such as A, C, and E), minerals, dietary fiber, polyunsaturated fatty acids, and natural bioactive compounds (NBCs), in which the MD is rich [17]. NBCs, such as polyphenols, are described as organic compounds of various origins (mainly from plant-based foods) and with different chemical structures, which exert beneficial actions on the organism and protect against the onset of chronic degenerative non-communicable diseases, such as cardiovascular diseases and obesity [18].

EVOO is an MD cornerstone food and a good source of NBCs, such as oleuropein, tyrosol, and hydroxytyrosol (HTyr). The latter exert several beneficial effects, such as anti-inflammatory, antioxidant, hypolipidemic, hypoglycemic, and cardioprotective effects [19].

In this regard, the PREDIMED study, involving individuals at high cardiovascular risk, showed that the incidence of major cardiovascular events was lower among those who followed an MD, supplemented with EVOO or nuts, compared to those who followed a reduced-fat diet [20]. The consumption of EVOO, along with that of plant-based foods, increases the amounts of other polyphenol compounds, such as chlorogenic acid and rutin. Several studies have shown that rutin is capable of (i) lowering blood glucose, (ii) regulating insulin secretion, (iii) counteracting lipid accumulation, (iv) exerting anti-inflammatory effects, and (v) restoring gut microbiota eubiosis [21,22,23,24].

The MD consists of a wide range of both animal and plant-based foods, rich in N-acylethanolamines (NAEs), such as palmitoylethanolamide (PEA), which is an endocannabinoid-like bioactive lipid mediator. Especially when used in formulations with enhanced oral bioavailability (like micronized or ultramicronized), PEA was shown to counteract hepatic metabolic inflexibility, reprogramming mitochondrial function and efficiency [25] to positively modulate the microbiota composition [26] and to promote white-to-beige adipose tissue conversion [27].

Recently, the effects of a formulation containing co-micronized PEA and rutin (mPEA–rutin), associated with HTyr, have been investigated in a murine model of obesity-induced metabolic alterations. The authors highlighted a reduction in fat mass after the administration of this formulation, as well as its ability to counteract glucose and lipid dysmetabolism in mice [28].

However, to date, no clinical studies have examined this formulation’s metabolic alterations in humans. The purpose of this randomized crossover double-blind placebo-controlled pilot study was to evaluate the potential beneficial effects of combining a tailored calorie-controlled MD with a natural and patented food supplement (FS), containing mPEA–rutin and HTyr, in patients with a diagnosis of MetS according to the IDF criteria.

## 2. Materials and Methods

### 2.1. Design of the Study

**Figure 1:**
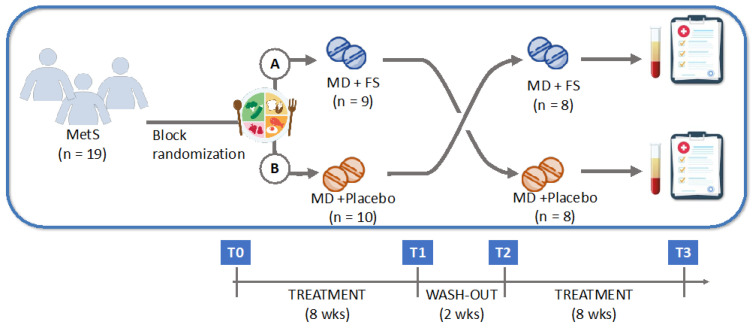
Study design. Abbreviations: MD, Mediterranean diet; MetS, metabolic syndrome; FS, food supplement.

The pilot study was a randomized crossover double-blind placebo-controlled study. The enrolled patients were block-randomized to the eight-week administration of the FS or a placebo. After a two-week washout period, the treatments were reversed. All patients received a tailored calorie-controlled MD during the study. The time points of the study were as follows (Figure 1).

- From T0 to T1, patients were randomized to the FS or placebo. In particular, they followed a tailored calorie-controlled MD and took two indistinguishable capsules (cps) of the respective treatment every day for eight weeks.
- From T1 to T2 (two-week period), patients received no treatment, except the MD.
- From T2 to T3, patients received the reverse treatment compared to the T0-T1 phase.

At baseline, all patients were subjected to a detailed physiological, remote pathological, and upcoming pathological anamnesis. They also underwent a detailed nutritional anamnesis in order to provide each patient with a tailored calorie-controlled MD. Laboratory parameters and those detected by capillary sampling, anthropometry, body composition analysis, ultrasound examination, blood pressure (BP) monitoring, the 36-Item Short-Form Health Survey (SF-36) questionnaire, the handgrip strength test (HGST), and physical performance evaluations of the patients were assessed at each time point.

### 2.2. Study Population

The study population consisted of 19 patients with MetS (average age of 65 ± 10 years), consisting of 11 females and 8 males. The patients were enrolled at the U.O.C. of Internal Medicine—Center for Hypertension and Geriatrics of the University Hospital of Rome Tor Vergata.

The inclusion criteria were as follows:

- patients with MetS, according to the IDF guidelines (for the Europids): waist circumference ≥ 94 cm (in men) or ≥88 cm (in women) plus at least two of the following criteria: triglycerides ≥ 150 mg/dL (or treatment with hypotriglyceridemic drugs), high-density lipoprotein (HDL) cholesterol < 40 mg/dL (men) or <50 mg/dL (women) (or treatment with hypolipidemic drugs), BP ≥ 130 mm/85 mmHg (or treatment with antihypertensive drugs), and fasting plasma glucose (FPG) ≥ 100 mg/dL (or a T2DM diagnosis) [29];
- age between 18 and 80 years;
- both sexes.

The exclusion criteria were as follows:

- cancer in the active phase;
- positivity for human immunodeficiency virus (HIV), hepatitis B surface antigen (HBsAg), or hepatitis C virus (HCV);
- inflammatory or infectious diseases in the acute phase;
- intake of PEA- and HTyr-based FS and/or other antioxidants 3 months prior to the study;
- refusal to sign informed consent form.

The experimental protocol, which complied with the 1975 guidelines of the Declaration of Helsinki, was approved by the Independent Ethical Committee of Policlinico Tor Vergata (protocol code R.S. 262.22, 20 December 2022).

### 2.3. Characteristics of Tailored Calorie-Controlled Mediterranean Diet

The calorie-controlled MD was developed by nutritional biologists and tailored to the clinical features of the patients. The prescribed MD was the same in both phases of the study, namely the MD + FS period and MD + placebo period. The following daily macronutrient requirements were applied: 45–60% of carbohydrates, 20–35% of lipids, and 10–12% of protein (Table 1). Regarding micronutrients, they were in line with the nutritional requirements estimated by the latest Italian LARN (Reference Intake of Nutrients and Energy for Italian Population) [30]. The Harris–Benedict formula was used to calculate each patient’s basal metabolic rate (BMR). The daily energy requirements (DER, Kcal) of the patients were calculated by multiplying the BMR by the physical activity level (PAL). Based on the DER, a personalized calorie restriction was applied to each patient (approximately 500 kcal), with the DER maintained above the patient’s BMR. The macronutrient percentages and the food’s quality were determined according to each patient’s comorbidities and his/her clinical characteristics. The lower end of the carbohydrate range (45–50%) was considered for patients who presented an FPG ≥ 100 mg/dL, hypertriglyceridemia, and current hypoglycemic and/or hypotriglyceridemic drug treatment, as well as considering the food’s glycemic index. Regarding the protein percentage, a protein-controlled MD was formulated only for those patients who were affected by chronic kidney disease, namely characterized by protein intake equal to 0.8 g/kg desirable b.w./day. In this case, whole foods were excluded and attention was paid to the consumption of other foods, according to electrolyte imbalances [31,32,33]. For other patients, protein intake of 1.0–1.2 g/kg desirable b.w./day was considered. Regarding lipids, the consumption of raw EVOO provided the main source of fats.

The adherence to the tailored calorie-controlled MD was evaluated through a food diary. One week before each study time point (namely T1, T2, and T3), the patients filled out a food diary, noting all foods, condiments, and drinks consumed during the day, from breakfast to dinner, including snacks. The food diary was brought by the patient to each check-up visit in order to monitor their adherence to the prescribed nutritional plan.

### 2.4. Characteristics of the Food Supplement

The tested FS was composed of mPEA–rutin and HTyr as active ingredients. In particular, one cps of FS contained 360 mg of mPEA–rutin (particle size of 2.0–10.0 microns) and 15 mg of HTyr (Normast^®^ 3, Epitech Group SpA, Saccolongo, Padova, Italy). The cps of the FS and placebo were indistinguishable; they had a diameter of 12.5 mm and a weight of 600 mg and they were both coated with an orange filming agent.

### 2.5. Laboratory Parameters and Capillary Blood Sampling

Regarding laboratory parameters, the blood count, FPG, renal function biomarkers (creatinine, estimated glomerular filtration rate, azotemia, uricemia), electrolytes (sodium, potassium, phosphorus, magnesium, calcium, and chlorine), blood iron level, lipid profile (such as total cholesterol, low-density lipoprotein (LDL) cholesterol, HDL cholesterol, and triglycerides), and inflammation biomarkers (C-reactive protein (CRP), erythrocyte sedimentation rate (ESR), and interleukin (IL-6)) were monitored at each time point of the study. The laboratory parameters were analyzed using an immunoturbidimetric or chemiluminescence method by electrochemiluminescence (Abbott Alinity Series Instrument, Milan, Italy), except for IL-6 (reference range: 0–50 pg/mL), which was determined by the chemiluminescence method (IMMULITE 2000 instrument, Siemens, Milan, Italy). All parameters were analyzed according to standard procedures in the clinical chemistry laboratories of the University Hospital of Rome Tor Vergata.

Capillary samplings were used to evaluate oxidative stress (free oxygen radical test (FORT)) and the antioxidant defenses (free oxygen radical defense (FORD)), which were analyzed with the CR4000 photometer (Callegari 1930, Parma, Italy). The FORT and FORD, through various solution-catalyzed reactions, were used to determine the concentrations of reactive oxygen species (ROS) and antioxidant compounds, respectively. One FORT unit corresponds to 0.26 mg/L hydrogen peroxide (H_2_O_2_). Concerning the FORT, test values below 300 U were considered “optimal” and those between 300 and 330 U “borderline”, while values above 330 U indicated the presence of oxidative stress. Regarding the FORD, values above 1.53 mmol/L Trolox equivalents were considered “optimal”, those between 1.53 and 1.07 mmol/L Trolox equivalents were “borderline”, and those below 1.07 mmol/L Trolox equivalents were “low” [34].

### 2.6. Measurement of Anthropometric Parameters and Body Composition Assessment

The body weight (kg) and stature (m) of the enrolled patients were collected by using a Seca scale with a built-in stadiometer (model 700, Hamburg, Germany). The measurements were recorded to the nearest 0.01 kg for body weight and 0.1 cm for stature. The circumference of the waist, abdomen, and hips was assessed using a Seca measuring tape (model 201, Hamburg, Germany).

The body mass index (BMI) of the patients was calculated by dividing the body weight by the stature squared (kg/m^2^). A bioelectric impedance analysis (BIA) was performed through the use of the EGF Plus^®^, based on the bioelectrical impedance vector analysis (BIVA) technology, at a 50 kHz frequency. The software Bodygram HBO ver. 1.31 (Estor, Pero, MI, Italy) was used to assess the patients’ body compositions, and the following parameters were recorded: total body water (in L and %), extracellular water (ECW) (in L and %), intracellular water (ICW) (in L and %), fat mass (FM) (in kg and %), fat-free mass (FFM) (in kg and %), body cell mass (BCM) (in kg and %), body cell mass index, BMR (Kcal), phase angle (PhA), resistance (Ω), and reactance (Ω).

### 2.7. Ultrasonographic Examination

To detect muscle mass (MM) changes, all patients underwent an ultrasound examination of the quadriceps rectus femoris thickness (QRFT) at 12 (the midpoint between the antero-superior iliac spine and the upper limit of the patella) and at 23 (the boundary point between the lower third and the upper two-thirds of the quadriceps muscle). This ultrasound technique is considered an innovative diagnostic tool capable of reflecting the patient’s total MM. The ultrasonographic evaluation was carried out by the same operator (A.N.), with 20 years’ experience, and with the ultrasound equipment Esaote MyLab70 XVision (Genova, Italy) and the linear probe LA523, through B-mode modulation with a 7.5 MHz transducer. In order to perform the ultrasonographic evaluation, three measurements were recorded bilaterally for both muscle anatomical landmarks, in the supine position and with both knees in extension. The mean value of the three measurements was reported. The probe was placed perpendicularly to the long axis of the muscle, covered with an abundant gel layer, on which minimal external pressure was exerted in order to prevent its compression [35,36].

### 2.8. Blood Pressure Monitoring

The systolic and diastolic BP and the heart rate were monitored with an automatic sphygmomanometer. The arm cuff was located at the heart level and only the third of the three performed measurements was recorded [37].

### 2.9. SF-36 Questionnaire

The SF-36 questionnaire was used to assess the health-related QoL of the patients. It consists of 36 items divided into nine spheres: perception of general health (5 questions), health changes (1 question), physical functioning (10 questions), perception of pain (2 questions), social functioning (2 questions), emotional well-being (5 questions), fatigue (4 questions), activity limitations due to health status (4 questions), and activity limitations due to emotional state (3 questions). For each sphere, it is possible to obtain values between 0 and 100, which directly correlate with the patient’s psychophysical well-being [38].

### 2.10. Evaluation of Muscle Strength and Physical Performance

Muscle strength was analyzed through the HGST, a dynamometer that assesses the handgrip force (Jamar Plus, Performance Health-Warrenville, IL, USA). The seated patients were asked to squeeze the dynamometer as hard as possible with the elbow of the working hand at 90° close to the hip. The HGST was performed three times with both limbs alternately and the average value was considered. The pathological cut-offs of the HGST are <30 kg for men and <20 kg for women [39].

Physical performance was evaluated through three tests.

- The short physical performance battery, a series of exercises including the gait speed (4 m walking), power (five-times chair sit to stand), and balance (tandem test). Each battery is scored up to 4 points and their sum indicates the level of performance. A total of 12 points is the best score [40].
- The stair climb power test, which analyzed the power of the lower limbs. Patients had to climb ten steps as rapidly as possible, without running or jumping. The time required to complete the test was recorded [41].
- The six-minute walk test, which evaluated the functional capacity of the patients. They had to walk, without running, for six minutes on thirty meters of flat corridor. At the end of the test, the walk distance was recorded [42].

### 2.11. Statistical Analysis

Data analysis was performed using the generalized linear mixed model (GLMM), adjusted to account for the interaction between the treatment and administration order (i.e., FS first or reversed after washout). Therefore, besides the covariates “sex” and “age”, the treatment sequence (i.e., FS/placebo or placebo/FS) was added. The interaction between the time and sequence was also evaluated to adjust for possible carryover effects. Values are reported as the mean ± standard error of the mean or standard deviation, as specified. A *p*-value ≤ 0.05 was considered statistically significant.

This manuscript reports the preliminary data analysis performed on approximately 30% of the entire sample involved in the study. Further analyses and investigations (e.g., post hoc analyses) will be performed once the study has been completed.

## 3. Results

The anthropometric and epidemiological features of the enrolled population at study entry are reported in Table 2.

Three patients dropped out between T2 and T3 for reasons unrelated to the treatment. Therefore, 16 patients with MetS completed the whole study.

**Figure 2:**
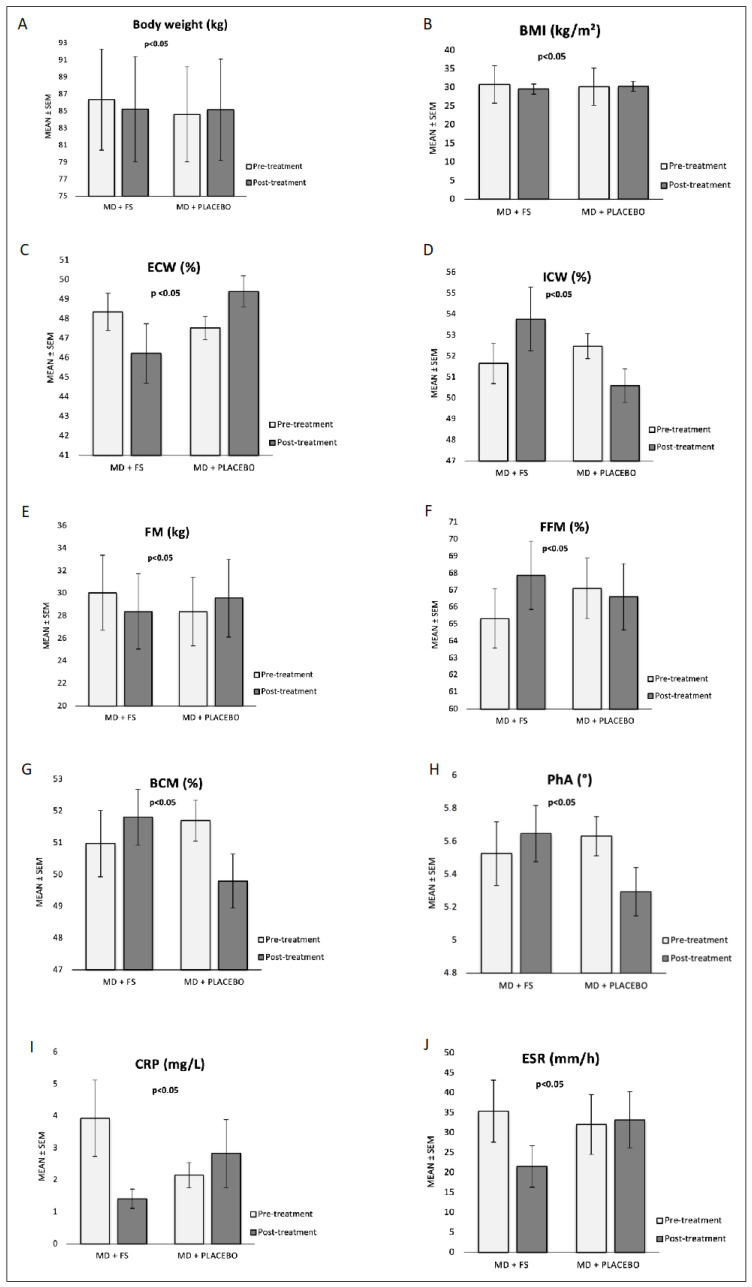
The histograms illustrate the pre- and-post-treatment parameters (after eight weeks) showing a significantly different response between the two groups. Abbreviations: BCM, body cell mass; BMI, body mass index; CRP, c-reactive protein; ECW, extracellular water; ESR, erythrocyte sedimentation rate; FFM, fat free mass; FM, fat mass; ICW, intracellular water; PhA, phase angle.

The effects observed in patients under the FS or placebo, irrespective of the treatment sequence, are reported below. As shown in Figure 2A,B, a statistically significant difference between the groups was observed over time in the body weight (*p* < 0.05) and BMI (*p* < 0.05), with an albeit small decrease in response to the FS (pre–post treatment body weight, 86.36 ± 5.94 kg vs. 85.25 ± 6.18 kg; pre–post treatment BMI, 30.83 ± 1.31 kg/m^2^ vs. 29.62 ± 1.32 kg/m^2^), compared to a slight increase after placebo administration (body weight, 84.62 ± 5.59 kg 85.16 ± 5.96 kg; BMI, 30.28 ± 1.23 kg/m^2^ vs. 30.34 ± 1.35 kg/m^2^). No statistically significant differences were observed for the waist, abdomen, and hip circumferences.

Concerning the BIA, a statistically significant difference between the groups emerged in the ECW expressed as a percentage (*p* < 0.05) and in the FM expressed in kg (*p* < 0.05), with a decrease observed in response to the FS (pre–post treatment ECW 48.35 ± 0.96% vs. 46.23 ± 1.51%; pre–post treatment FM, 30.04 ± 3.33 kg vs. 28.38 ± 3.35 kg) and an increase after placebo administration (ECW, 47.52 ± 0.59% vs. 49.40 ± 0.80%; FM, 28.37 ± 3.05 kg vs. 29.58 ± 3.46 kg). The data are presented in Figure 2C,E.

In addition, a statistically significant difference between the treatment groups was also observed in the ICW expressed as a percentage (*p* < 0.05) and in the FFM expressed as a percentage (*p* < 0.05), as reported in Figure 2D,F. In particular, both the ICW (pre–post treatment 51.65 ± 0.96% vs. 53.77 ± 1.51%) and FFM (pre–post treatment 65.32 ± 1.74% vs. 67.87 ± 2.01%) increased in response to the FS, while they decreased after placebo administration (pre–post treatment ICW, 52.48 ± 0.59% vs. 50.60 ± 0.80%; pre–post treatment FFM, 67.11 ± 1.78% vs. 66.61 ± 1.96%).

Similarly, the pre–post treatment change in the BCM expressed as a percentage (Figure 2G) and PhA (Figure 2H) showed statistically significant differences between the groups (BCM, *p* < 0.05; PhA, *p* < 0.05). Similarly, both parameters increased in response to the FS (pre–post treatment BCM, 50.97 ± 1.04% vs. 51.81 ± 0.88%; pre–post treatment PhA, 5.53 ± 0.19° vs. 5.65 ± 0.17°) and decreased following placebo administration (BCM, 51.69 ± 0.64% vs. 49.79 ± 0.85%; PhA, 5.63 ± 0.12° vs. 5.29 ± 0.15°). No relevant differences were observed between the groups for any other BIA-estimated measure.

The pre–post treatment changes in the ESR showed a statistically significant difference between the groups (*p* < 0.05, Figure 2I), with a clear decrease in response to the FS (pre–post treatment 35.42 ± 7.79 mm/h vs. 21.56 ± 5.21 mm/h) and a slight increase after the placebo (pre–post treatment, 32.06 ± 7.49 mm/h vs. 33.22 ± 7.08 mm/h). Similarly, the changes over time in CRP were statistically different between the groups (*p* < 0.05, Figure 2J), with a marked decrease in response to the FS (pre–post treatment, 3.93 ± 1.19 mg/L vs. 1.41 ± 0.30 mg/L) and an albeit small increase following the placebo (pre–post treatment 2.15 ± 0.39 mg/L vs. 2.83 ± 1.06 mg/L).

Furthermore, although the difference between the groups did not reach statistical significance, the IL-6 plasma levels showed a trend reduction over time after the FS was administered (pre–post treatment 8.0 ± 1.73 pg/mL vs. 4.8 ± 1.20 pg/mL) but not after the placebo was administered (pre–post 5.6 ± 0.94 pg/mL vs. 5.01 ± 1.07 pg/mL).

The two-week washout period was sufficiently long to eliminate carryover effects.

The analysis of the data on the laboratory parameters and those related to the BP, capillary sampling, anthropometrics (body circumferences), ultrasound examination of the QRFT (Table 3), SF-36 questionnaire, physical performance, and muscle strength showed no statistical differences between the groups.

Finally, none of the patients reported adverse events or showed clinically relevant deviations in the routine blood analysis throughout the study.

Results are represented as the mean ± SEM. Abbreviations: BCM, body cell mass; BMI, body mass index; CRP, C-reactive protein; ECW, extracellular water; ESR, erythrocyte sedimentation rate; FFM, fat-free mass; FM, fat mass; FS, oral food supplement; ICW, intracellular water; MD, Mediterranean diet; *p*, *p*-value, PhA, phase angle; S.E.M.; standard error of the mean.

## 4. Discussion

Visceral obesity is a key feature of adiposopathy in MetS, which is a complex disorder characterized by the simultaneous presence of several metabolic risk factors [43].

Metabolic alterations, such as obesity and MetS, can compromise the hypothalamic regulatory function in the brain, responsible for the maintenance of energetic homeostasis, including food intake and energy expenditure. In patients with MetS, there is an increase in the circulating levels of the anorexigenic hormone leptin, produced by the adipose tissue; however, due to leptin resistance, the inhibitory effect of leptin on neurons, expressing neuropeptide Y and agouti-related protein (AgRP), does not occur, thus resulting in an increased hunger stimulation response. A central role in this effect is also played by the action of the orexigenic hormone ghrelin, through the positive stimulation of the same neurons. On the other hand, a reduction in the gut-derived satiety peptides is observed, generating, consequently, impaired satiety stimulation by neurons expressing proopiomelanocortin (POMC) and cocaine- and amphetamine-regulated transcript (CART) [44]. Consequently, it is a considerable challenge for patients with MetS to achieve an ideal body weight or to maintain weight loss through a calorie-controlled diet [45], because the body seems to retain an obesogenic memory to defend itself against weight changes [46].

In accordance with the diagnostic criteria defined by the IDF for Europids, abdominal obesity was used as the first diagnostic criterion to select the patients for our randomized crossover double-blind placebo-controlled pilot study. The other two most relevant criteria were a diagnosis of arterial hypertension (AH) and low HDL cholesterol levels. In fact, 100% of them presented a diagnosis of AH, while 89.5% had low HDL cholesterol levels and/or were being treated with hypolipidemic drugs. A similar percentage has been found for patients with hypertriglyceridemia and/or in treatment with hypotriglyceridemic drugs (84.2%), a considerably higher value than those with an impaired fasting glucose (IFG) or a T2DM diagnosis (47.4%). Therefore, at baseline, our study population consisted of 19 patients with first-degree unhealthy metabolic obesity.

It is known that unhealthy and unsustainable dietary habits and a sedentary lifestyle are very often at the root of obesity [47]. For this reason, “eradicating” these unhealthy behaviors was the main focus of our study, “sowing” in the patients the key principles of nutritional education, such as those of the MD, and evaluating the possible beneficial effects of combining the MD with an FS based on mPEA–rutin and HTyr as active ingredients.

However, after the eight-week MD + placebo period, the MD alone did not appear sufficient to improve, in a statistically significant manner, any of the parameters analyzed in the study. The food diaries filled out by patients showed high adherence to the prescribed MD. However, since these are preliminary data, we believe that the sample size is currently insufficient to detect significant changes in all parameters investigated in the study.

In the study conducted by Shai et al., 322 patients with first-degree obesity were subjected to a moderate-fat and restricted-calorie MD and were evaluated for two years. The authors highlighted that the MD led to the greatest weight reduction during the first six months; this period was followed by a maintenance phase of partial rebound and a plateau, with a final weight loss of 4.4 kg after twenty-four months. The effects of MD on the reductions in the waist circumference, systolic and diastolic BP, triglycerides, and FPG and on the increase in HDL cholesterol were observed to be superior at twenty-four months compared to six [48].

As for the loss of body weight and the enhancement of MetS comorbidities, they cannot be attributed solely to healthy eating habits. In fact, obesogenic memory, as previously mentioned, also depends on a lack of physical activity [49], sleep deprivation, stress, disrupted circadian rhythms (clock genes) [50], and drug consumption (such as beta-blockers, antipsychotics, glucocorticoids, and antidepressants) [51].

In fact, in our study, the effects of the tailored calorie-controlled MD were statistically significant when the diet was supplemented with the FS, rather than with the placebo. Therefore, our results suggest that the improvements in the outcomes of the patients after the MD + FS periods were attributable to the combined treatment, in which we could also observe the beneficial action induced by mPEA–rutin and HTyr, and not only that derived from the MD. At the end of an eight-week treatment with the FS, we observed an improvement in the anthropometric parameters, such as body weight and BMI. Moreover, through the BIA, we observed a reduction in the ECW (in %) and FM (in Kg) and an increase in the ICW (in %), FFM (in %), BCM (in %), and PhA (°), which were statistically significant, compared to the MD + placebo period.

To date, only preclinical studies have evaluated the action of PEA on the adipose tissue. In the study conducted by Annunziata et al., an ultramicronized formulation of PEA (umPEA) promoted the conversion of energy-storing white adipocytes into energy-consuming brown ones. The authors concluded that umPEA possesses metabolic, thermogenic, and anti-inflammatory effects on the adipose tissue, thus contributing to body weight and fat mass loss, to the overcoming of leptin resistance, and to the “reprogramming” of adipose homeostasis [27]. Moreover, Mattace Raso and colleagues demonstrated that umPEA also causes a reduction in food intake through two mechanisms that could explain its effect on FM loss. The first acts at the peripheral level on the adipose tissue, through the normalization of leptin synthesis, while the second operates at the central level on the hypothalamus, through the normalization of leptin sensitivity and the modulation of POMC and AgRP, which negatively regulate food consumption [52]. These mechanisms could therefore explain the significant weight loss and the improvement in body composition observed in our study population after the MD + FS period. However, no reduction in the circumference of the abdomen was seen after an eight-week treatment, thus highlighting the need for a longer treatment period. The FS’ effects on the improvement in the body composition can also be attributed to the action of HTyr, the major polyphenolic compound present in EVOO. Both pre-clinical and clinical studies have highlighted the beneficial actions of this NBC in the prevention of high-fat-diet-induced obesity [53] and a reduction in body weight, as well as visceral FM [54].

In both the FS- and placebo-supplemented arms, the mean pre-treatment levels of inflammation biomarkers were either within (i.e., CRP) or above (i.e., ESR) the normal ranges. Interestingly, the eight-week intervention resulted in a sharp decrease in response to the FS but not to the placebo, with a statistically difference between the two arms. Patients with MetS are characterized by a chronic state of low-grade inflammation, named meta-inflammation, which is observed in all tissues involved in energy homeostasis [2]. The effects of PEA on meta-inflammation over time have not been deeply investigated yet. The pre-clinical study conducted by Lama et al., in an animal model, showed that the seven-week daily administration of umPEA (30 mg/kg b.w.) reduced pro-inflammatory mediators like tumor necrosis factor (TNF)-α, IL-1β, monocyte chemoattractant protein-1, and lipopolysaccharide [55].

Furthermore, a five-week period of PEA administration, in a murine model of hyperphagia, was found to reduce macrophage infiltration in the white adipose tissue and the production of pro-inflammatory cytokines [52].

The effects of HTyr and rutin on meta-inflammation have been better elucidated in the literature. Both these NBCs are present in EVOO [56,57], and they exert powerful anti-inflammatory and antioxidant effects due to their ability to scavenge ROS. Thanks to these properties, in 2011, the European Food Safety Authority recognized the health benefits of HTyr. The daily intake of 20 g of EVOO, containing at least 5 mg of HTyr and of its derivatives (e.g., oleuropein complex and tyrosol), is able to reduce oxidative stress, to exert antioxidant effects, and to protect body cells and LDL from oxidative damage [58]. This may explain the preventive properties of EVOO against chronic degenerative diseases [19,59,60]. The reduction in inflammatory biomarkers observed here may thus be related to either mPEA–rutin and HTyr or to the combination of the two compounds.

With regard to the other diagnostic criteria of MetS, we did not observe a statistically significant reduction in FPG, triglycerides, or BP or an increase in HDL cholesterol levels after the MD + FS period, compared to the MD + placebo one. However, only after the MD + FS period, we observed a trend towards a reduction in FPG, BP, and triglycerides. Although HTyr exerts hypolipidemic, hypoglycemic, and cardioprotective effects [19], and rutin possesses antihyperglycemic ones [60], we attribute the lack of a significant change in these parameters to the previous drug therapy. In fact, at baseline, both groups had average values of the four parameters mentioned above that were within the normal range, and only 47.4% of them had IFG or a T2DM diagnosis.

The present clinical study is currently ongoing. A limitation of our preliminary study was the small sample size, while the strengths were the study design and close monitoring of the enrolled patients.

## 5. Conclusions

MetS is a complex metabolic disorder requiring a multidisciplinary approach and involving different health professionals, such as medical specialists and those involved in lifestyle modification, such as nutritionists, clinical kinesiologists, and psychologists. Patients with MetS need to be motivated and supported through visits with close follow-up and with constant monitoring of dietary interventions. In fact, although, in our clinical study, the MD alone for eight weeks was not able to elicit clinically significant changes in patients, we strongly believe that a tailored nutritional adjuvant therapy should be combined with drug treatment. These adjuvant therapies also include those based on FSs. Although, in MetS, the beneficial effects of HTyr have already been investigated in the literature [61], for the first time, in our pilot study, we have investigated the potential effects of the combination of HTyr and mPEA–rutin in these patients. After an eight-week treatment with two cps/day of the FS, in combination with a tailored calorie-controlled MD, we observed a significant reduction in body weight and BMI, an improvement in body composition parameters detected by BIA (such as FFM, FM, PhA, BCM, ECW, and ICW), and a reduction in inflammatory biomarkers (like ESR and CRP). Our randomized crossover double-blind placebo-controlled pilot study confirms the previous pre-clinical data, namely that HTyr and mPEA–rutin are molecules of natural origin with powerful activity against adiposopathy and “sick fat” formation.

## Figures and Tables

**Table 1:** Average bromatological composition of the tailored calorie-controlled Mediterranean diet formulated for patients.

Macronutrients		
	%	g
Protein	16.72	
Lipids	28.57	
Carbohydrates	53.29	
Fiber		23.63

**Table 2:** Anthropometric and epidemiological features of the study population.

Number of MetS Patients: 19			
Study Population Features		MetS Patients With (%)	
Age (years)	65 ± 10 *	Abdominal obesity	100
Gender (F/M)	11/8	HTG (or hypotriglyceridemic drugs)	84.2
Weight (kg)	86.7 ± 25.3 *	Low HDL-C (or hypolipidemic drugs)	89.5
BMI (kg/m^2^)	31 ± 5.5 *	AH (or antihypertensive drugs)	100
Abdominal circumference (cm)	109.2 ± 16.8	IFG (or previously diagnosed T2DM)	47.4

* Data expressed as mean ± standard deviation. Abbreviations: AH, arterial hypertension; BMI, body mass index; F, female; HDL-C high-density lipoprotein cholesterol; HTG, hypertriglyceridemia; IFG, impaired fasting glucose; M, male; MetS, metabolic syndrome; T2DM, type 2 diabetes mellitus.

**Table 3:** Parameters of particular interest that did not show statistically significant differences between the treatment arms.

	MD + FS		MD + PLACEBO		
Laboratory Parameters	Pre-Treatment	Post-Treatment	Pre-Treatment	Post-Treatment	*p*-value
Triglycerides (mg/dL)	109.0 ± 13.9	101.2 ± 11.6	119.9 ± 17.2	121.0 ± 20.5	n.s.
Total cholesterol (mg/dL)	173.3 ± 10.1	170.7 ± 9.5	179.7 ± 10.9	177.5 ± 9.4	n.s.
HDL cholesterol (mg/dL)	48.7 ± 3.5	48.4 ± 3.6	47.5 ± 3.7	47.4 ± 3.8	n.s.
LDL cholesterol (mg/dL)	110.8 ± 8.3	105.5 ± 9.0	111.3 ± 8.5	104.7 ± 7.6	n.s.
FPG (mg/dL)	99.7 ± 8.9	92.8 ± 4.7	97.9 ± 8.5	101.9 ± 8.7	n.s.
IL-6 (pg/mL)	8.0 ± 1.7	4.8 ± 1.2	5.6 ± 0.9	5.1 ± 1.1	n.s.
Blood Pressure Parameters	Pre-Treatment	Post-Treatment	Pre-Treatment	Post-Treatment	*p*-value
SBP (mmHg)	124.4 ± 2.8	122.4 ± 3.7	128.2 ± 2.8	127.2 ± 3.5	n.s.
DBP (mmHg)	72.3 ± 2.2	70.1 ± 1.8	73.3 ± 1.6	72.6 ± 1.9	n.s.
Capillary Sampling Parameters	Pre-Treatment	Post-Treatment	Pre-Treatment	Post-Treatment	*p*-value
FORT (U)	350.4 ± 30.3	352.2 ± 28.7	372.7 ± 27.1	354.0 ± 20.3	n.s.
FORD (mmol/L Trolox equivalents)	1.50 ± 0.1	1.33 ± 0.1	1.41 ± 0.1	1.33 ± 0.1	n.s.
Anthropometric Parameters	Pre-Treatment	Post-Treatment	Pre-Treatment	Post-Treatment	*p*-value
WC (cm)	97.4 ± 3.6	95.9 ± 3.9	98.7 ± 3.7	96.9 ± 3.6	n.s
AC (cm)	106.2 ± 4.0	105.3 ± 4.3	105.3 ± 2.8	106.6 ± 3.6	n.s.
HC (cm)	111.7 ± 3.7	108.9 ± 4.0	111.7 ± 3.5	109.4 ± 3.6	n.s.
Ultrasound Examination Parameters	Pre-Treatment	Post-Treatment	Pre-Treatment	Post-Treatment	*p*-value
QRFT 12 right (cm)	1.7 ± 0.1	1.9 ± 0.1	1.8 ± 0.1	1.8 ± 0.1	n.s.
QRFT 23 right (cm)	1.7 ± 0.05	1.8 ± 0.1	1.7 ± 0.1	1.7 ± 0.1	n.s.
QRFT 12 left (cm)	1.8 ± 0.1	2.0 ± 0.1	1.8 ± 0.1	1.9 ± 0.1	n.s.
QRFT 23 left (cm)	1.7 ± 0.05	1.8 ± 0.1	1.7 ± 0.1	1.7 ± 0.1	n.s.

The data are reported as the mean ± standard error of the mean. Abbreviations: AC, abdominal circumference; DBP, diastolic blood pressure; FORD, free oxygen radical defense; FORT, free oxygen radical test; FPG, fasting plasma glucose; FS, food supplement; HC, hip circumference; HDL, high-density lipoprotein; IL-6, interleukin-6; LDL, low-density lipoprotein; MD, Mediterranean diet; n.s., not significant; QRFT, quadriceps rectus femoris thickness; SBP, systolic blood pressure; WC, waist circumference.

## Data Availability

The datasets presented in this article are not readily available because it contains preliminary and personal patients’ data. Requests to access the datasets should be directed to the corresponding authors.
